# Young Sexual Minority Adolescent Experiences of Self-expression and Isolation on Social Media: Cross-sectional Survey Study

**DOI:** 10.2196/26207

**Published:** 2021-09-15

**Authors:** Linda Charmaraman, Rachel Hodes, Amanda M Richer

**Affiliations:** 1 Wellesley Centers for Women Wellesley College Wellesley, MA United States

**Keywords:** social media, social networking sites, sexual minorities, cyberbullying, depression, loneliness, self-harm, social support, adolescents

## Abstract

**Background:**

Early adolescent years are marked by pervasive self- and peer-regulation regarding gender and sexuality norms, which can affect the mental well-being of sexual minority youth. During this developmental period, social media use is also emerging as a dominant mode of communication with peers, allowing for both risk and resilient behaviors that can impact well-being.

**Objective:**

This exploratory study aims to examine how sexual minorities in middle school use social media, who they are connected to and for what purposes, and the associations between these behaviors and mental well-being compared with their heterosexual peers.

**Methods:**

In our cross-sectional survey study of 1033 early adolescents aged between 10 and 16 years (average age 12.7, SD 1.21 years) from 4 middle school sites in the Northeastern United States, we conducted an exploratory study comparing sexual minorities (212/873, 24.3% of sample with known sexual orientation) with their heterosexual peers (n=661), obtaining an 84.46% (1033/1223; total possible) response rate.

**Results:**

Sexual minorities reported having smaller networks on their favorite social media website (*β*=−.57; *P*<.001), less often responded positively when friends shared good news (*β*=−.35; *P*=.002), and less often tried to make friends feel better when they shared bad news (*β*=−.30; *P*=.01). However, sexual minorities more often reported joining a group or web-based community to make themselves feel less alone (*β*=.28; *P*=.003), unlike heterosexual youth. Sexual minorities had higher averages of loneliness and social isolation (*β*=.19; *P*<.001) than heterosexual students. Sexual minorities were also twice as likely to have tried to harm themselves in the past (*β*=.81; odds ratio [OR] 2.24, 95% CI 1.64-3.06; *P*<.001) and were more likely to have symptoms that reached the Center for Epidemiological Studies-Depression definition of depression (*β*=.15; OR 1.16, 95% CI 1.08-1.25; *P*<.001). About 39.1% (83/212) of sexual minorities had no one to talk to about their sexual orientation. Sexual minorities were 1.5 times more likely to have joined a social media website their parents would disapprove (*β*=.41; OR 1.50, 95% CI 1.14-1.97; *P*=.004) and more likely to report seeing videos related to self-harm (*β*=.33; OR 1.39, 95% CI 1.06-1.83; *P*=.02) on the web than heterosexual youth.

**Conclusions:**

Given previous reports of supportive and safe web-based spaces for sexual minority youth, our findings demonstrated that sexual minority youth prefer to maintain small, close-knit web-based communities (apart from their families) to express themselves, particularly when reaching out to web-based communities to reduce loneliness. Future longitudinal studies could determine any bidirectional influences of mental well-being and social media use in sexual minorities during this difficult developmental period.

## Introduction

### Background

Web-based communication opportunities via mobile devices, internet, and social media platforms remain a largely understudied context of sexual minority youth [1], defined in this study as youth who identify as gay, lesbian, bisexual, asexual, queer, questioning, or otherwise do not identify as heterosexual. Of these youth, 97% use at least one social media website. It is also known that adolescents are adopting social media at an earlier age, with YouTube, Instagram, and Snapchat surpassing Facebook as the most popular social media websites for youth aged from 13 to 17 years [2]. These web-based opportunities are particularly critical sources of risk and resilience to sexual minority youth given the disproportionate risks and limited access to social support they face in other contexts, such as home, school, and community [3]. Studies on sexual minority youths’ use of web-based communication technologies are rarely compared with their heterosexual peers. The Gay Lesbian Straight Education Network [4] analyzed data from 2010 and 2011 demonstrating that lesbian, gay, bisexual, transgender, or queer (LGBTQ) youth (aged 13-18 years, n=1960) spend about 45 minutes longer on the web on a daily basis than their heterosexual peers. A recent large study of 6309 youth aged 14-29 years found that less traditional sexual minority categories, including pansexual, asexual, queer, and gender nonconforming youth, are more likely to spend time on the web compared with other youth [1]. Scholars have theorized that sexual minority youth may be avid social media users because of frequent social exclusion in their offline lives [4]. More recent research has revealed that social media can also enhance the well-being of sexual minority youth by providing safe and accessible contexts to develop social support networks, such as exploring emerging sexual minority identities, locating and coming out to other sexual minority peers, and accessing identity-specific web-based resources [1].

### Early Adolescence as an Understudied Age Group

In the United States, the Federal Children’s Online Privacy Protection Act prevents individuals aged younger than 13 years from signing up for social media accounts, such as Facebook, Instagram, Snapchat, or TikTok [5]. Despite this regulation, a recent study estimated that almost half (49%) of adolescents aged 11 years or younger had a social media account, and by age 14, this increased to 85% [6]. In a recent review of social media research [7], which provided a summary of systematic reviews across 371 studies, the vast majority of studies examining the effects of social media focused on either young children or adults; when adolescents were investigated, the developmental stages within adolescence were not delineated. The authors of this review argue that early adolescence is still the most neglected developmental period, despite the fact that it is likely one of the most relevant and consequential times to examine relationships between social media and mental health. As adolescents transition biologically and socially into new school and digital environments, they are often most susceptible to peer influence effects, primarily because of increased identity exploration, more unsupervised time spent with peers, and the need for peer validation and approval [8].

The early adolescent years are also marked by pervasive self- and peer-regulation regarding gender and sexuality norms [9]. Peer influence and opinion are especially salient during this developmental period when youth experience more prejudicial and homophobic behaviors [10], making it an especially difficult time to come out as a sexual minority [11]. As societal attitudes change and LGBTQ individuals are more socially accepted, the average age of *coming out* has also shifted. Russell et al [12] noted a trend moving from an average coming-out age of 20 years in the 1970s to around 16 years in the 1990s to approximately 14 years in 2010 [13]. Despite the decades-ago trend that adolescents are coming out at younger ages, the vast majority of studies on sexual minorities and their mental health focus on older adolescents [1,4] and young adults in college years [1], as opposed to the early stages of self-realization, in which young adolescents must decide when and where to come out, both on the web and offline. Thus, the studies described below that used the terms *youth* or *young adults* most often refer to those aged 18 years and older, unless otherwise indicated.

### Sexual Minority Identity Development on the Web

Identity development in sexual minority populations refers to the process by which sexual minority adolescents learn to ascribe meaning and labels to their nonnormative experiences of sexuality [14,15]. As a new generation of youth come of age on the web, the internet serves as a major arena for forming and developing these identities. Fox and Ralston [16] found that participants in the process of LGBTQ identity formation often used social media as a tool to actively learn about themselves, their identities, and their communities, whether this engagement took the form of traditional information-seeking, experiential participation in the LGBTQ community, or observing the behaviors of LGBTQ role models on the internet. In a study of same-sex attracted youth aged from 14 to 21 years, Hillier and Harrison [17] also found that digital spaces served as arenas for discussing experiences of coming out, practicing same-sex dating, and forming friendships with other sexual minority youth, in relative safety compared with sexual minority youth’s offline environments.

Recent research has suggested a developmental trajectory wherein younger adolescents spend time in web-based LGBTQ communities exploring their identities through sharing content, microblogging, and participating in fandom activities using websites such as Instagram, whereas older adolescents and young adults use traditional networking websites such as Facebook to establish professional, romantic, and social connections branching out beyond their identity communities [1]. Web-based fandoms, which media scholars define as an “imagined and imaginative community” [18] based around the participatory consumption of popular culture, have been found to be a particularly salient form of web-based community for youth newly identifying as sexual minority. Fandom communities allow users to explore and experiment both inwardly and outwardly with their emergent identities, providing low-risk, protected web-based spaces where sexual minority youth are not required to connect their fandom identities with their *real* names or even commit themselves to a particular sexual identity label [19]. For example, sexual minority users might prefer to look at artwork with characters that resonate with their developing selves, or they might write stories related to their identity development to share within fandom communities.

### Social Media Networks, Settings, and Privacy

An ecosystem model has been suggested to understand the way in which many LGBTQ populations divide their time between different social media websites and categorize websites for different purposes depending on the affordances they offer regarding anonymity and content sharing [20]. Different platforms offer different opportunities for sexual minority youth to openly engage with identity-related content and form connections with peers. Tumblr, in particular, is a website that has been spotlighted for its ability to foster the queer community, in part because its design is not one of *default publicness* [21], and users can express themselves through multiple accounts and mediums, with less likelihood of being observed by offline networks [22]. These features are especially appealing to youth *in the closet* or developing a complex understanding of identity beyond what they would express to a primarily heterosexual audience.

Instagram, meanwhile, serves as a website of visual self-expression for many adolescents. It has been examined primarily as a platform where adolescents seek public social validation from their peer networks [23]. However, Instagram also has a particularly robust culture in which users create secondary accounts (called *Finstas*), which serve as private spaces on the internet where they can post illicit content, connect deeply with close friends, share more nuanced representations of themselves than might appear on their *real Instagrams*, and express negative emotions [24]. These features of Instagram use have not been examined through the lens of sexual identity but offer important insights into how adolescents negotiate privacy and self-presentation on websites that offer varying levels of visibility.

Research on how young adults approach the task of privacy management on various social media platforms has found that sexual minority and gender nonconforming youth face significant risks when disclosing their identity on the internet. The existence of *networked publics* means that youth active on the internet are often aware of the variety of audiences their content may be exposed to and develop strategies to make information legible only to audiences of their choosing [25]. Many adolescents aged as young as 13 years, although aware of the public nature of social media, are unwilling or disempowered to navigate complex privacy settings and find conflict between their desire to use a website and their inability to control the spread of their content on that website [26]. These issues can come to a head for LGBTQ youth, particularly when websites such as Facebook, which privileges single accounts and offline networks, lead young people to accidentally disclose their identities to unintended audiences, often with severe interpersonal and familial repercussions [21].

### Adolescent Loneliness, Social Support, and Web-Based Interactions

Scholars have theorized that as modern social media websites allow us to maintain connections electronically, finding contacts across unrestricted distances, and creating web-only friendships, there is a greater risk of losing physical social connections and inviting new types of social exclusion [27]. Researchers have also noted that adolescents are more physically isolated today than previous generations, who satisfy their need for social connection through physical relationships. Adolescents today are more comfortable meeting their social needs through computer-mediated communication [28]. Therefore, measuring perceived loneliness might be more nuanced among young people when web-based peer and offline relationships are most salient, and exploring their social identities is a prominent developmental task [29]. Loneliness has been challenging to define but is often accompanied by feelings of emptiness, anxiety, and social isolation [30]. According to the cognitive discrepancy model by Perlman and Peplau [31], loneliness is realized when an individual’s personal network of social relations is either quantitatively or qualitatively deficient; when they are disappointed with their expectations of interpersonal interactions, loneliness sets in. Studies have found that loneliness experienced in youth can have significant consequences for wellness [32], such as being more prone to problematic internet use [33] across multiple cultures or nationalities [34], poorer sleep quality, and lower immune response [35]. These wellness outcomes are of heightened concern for caregivers and practitioners of adolescents, especially while practicing social distancing during the COVID-19 pandemic.

For sexual minority youth, previous research has identified social support as the primary protective factor against negative mental health outcomes [36], including loneliness [37]. A large study (n=5542) of 13- to 18-year olds revealed that sexual minority adolescents believed that their internet friends were better than their in-person friends at providing social support [38]. Resilience, or patterns of positive adaptation to risk situations, has also been directly related to sexual minority youth’s sense of positive identity, which in turn reduces the likelihood of negative mental health outcomes [39]. An example of this resiliency can be found in sexual minority youth who use social networking websites to develop their sexual identity, who were found to have lower levels of paranoia than their heterosexual peers [37]. Alternatively, internalized homonegativity is related to mental health problems, such as depression and anxiety [40]. According to the minority stress theory [41], exposure to stressors such as discrimination, social rejection, and sometimes violence is a central cause of mental distress among sexual minorities. These distal stressors are related to proximal stressors such as expectations of rejection and internalized homonegativity. A strong social support system, particularly from the LGBTQ community, can help buffer the impact of these stressors on mental health outcomes. Although other web-based resources, such as e-therapies, have the potential to provide mental health support to sexual minority youth, many of these technologies have not been tailored to the LGBTQ experience and thus fail to address the specific challenges these adolescents face [42].

Sexual minority youth are significantly more likely to experience depressive symptoms, substance use, and truancy, particularly when faced with homophobic harassment [43], both on the internet and offline [44]. Gay Lesbian Straight Education Network [4] found that 42% of LGBTQ youth aged 13-18 years had been harassed on the internet, whereas 32% reported being sexually harassed on the internet; these percentages were significantly lower (15% and 8%, respectively) among non-LGBTQ youth. More recently, researchers have suggested that there is a relationship between the negative social media experiences of young sexual minority adults and their depressive symptoms [45]. Qualitative research has also examined the experiences of sexual minority youth with cyber-victimization, finding that it is often related to, although less common than, in-person harassment [38,46]. Across the many forms that cyber-victimization of sexual minority youth may take, they face particular vulnerability because of their often complex relationships with privacy, family, and closeted identity [47]. Sexual minority youth are also twice as likely as heterosexual youth to attempt suicide [48], particularly those who face family rejection [49]. Recent research analyzing Reddit posts classified as displaying loneliness showed that these posts were associated with future posting activity on the suicide subreddit threads [50]. This predictive modeling of social media activity can be useful when trying to understand how loneliness manifests itself through web searches and postings and when certain patterns in an adolescent’s social media behaviors put them at risk of self-harm or suicidality.

Due to the publicness, availability, and permanence of typical social media environments, peer influence effects can be heightened in terms of speed, volume, and scale, with adolescents sharing and viewing content with multiple networks within and outside their peer group [51,52]. Adolescents are socialized to adopt the web-based behaviors of their peers. For instance, their decisions about web privacy are shaped by peer norms regarding privacy settings [53]. Adolescents also tend to imitate their peers in the risky web-based content they post; for instance, adolescents who report more of their friends sharing alcohol references on social media are more likely to post about alcohol themselves [54]. Research has found that risky social media content can influence perceptions of peer norms as well, such as eating and weight-related attitudes and behaviors [55] and risky sexual norms [56]. Social media may encourage adolescents with mental health dilemmas to share their experiences with weight management or nonsuicidal self-injury on the internet as a way of receiving social support that is not accessible to them offline, which may reinforce or normalize such behaviors within some web-based communities [57]. Few studies have examined suicide contagion effects on social media, despite the existence of readily accessible self-injury content on platforms such as Instagram through the use of ambiguous hashtags [58] and Tumblr through communities that make visible and sometimes glorify self-harm practices [22]. There is a scarcity of research on the web-based peer influences of adolescent sexual minority populations, which may be similar or different from their heterosexual peers.

### Rationale for Exploratory Study

To date, there are no studies of sexual minority adolescents aged as young as 11 years (who are in the early stages of identifying as a sexual minority), their social media preferences and behaviors, potential influences of peer interactions, and how these preferences and web-based interactions are associated with their mental health. The vast majority of studies conducted on the effects of loneliness or social isolation focus on young or older adult populations; less is known about early adolescents, and studies on sexual minority youth are even more scarce. A recent review of studies on social media use, sexual minorities, and depression found that most studies broadly defined social media use, limiting their understanding to the quantity of time spent rather than analyzing the types of activities undertaken [59]. Literature on privacy management and socially supportive behaviors on social media often fails to incorporate the perspectives of sexual minority youth. Past studies have also focused on social networking platforms that young adults typically use, such as Facebook or Tumblr. This study included more current websites used by those in early adolescence, such as YouTube, TikTok, Snapchat, Instagram, and Discord.

### Objectives

This study has the following objectives:

1. Understand the differences and similarities between how early adolescent sexual minorities use social media compared with their heterosexual counterparts, including platforms, privacy settings, motivations for use, and secretive behaviors;
2. Investigate the web-based connections of early adolescent sexual minorities, whether they engage in socially supportive web-based behaviors, and whether they are motivated to connect with others to reduce social isolation compared with heterosexual peers;
3. Examine whether there are increased vulnerabilities in loneliness, depressive symptoms, and self-harm among early adolescent sexual minority social media users compared with their heterosexual counterparts.

## Methods

### Data Collection Procedures

Data were collected as part of a larger pilot and ongoing longitudinal survey of early adolescent social media use and behavioral health [60] since 2017. This human subject research study obtained institutional review board approval from Wellesley College. As there were no existing measures that had been piloted in our early adolescent population for key constructs related to social media use, the purpose of the mixed methods pilot study conducted in 2017-2018 was to integrate what past literature has indicated as key constructs and conduct key informant interviews with middle school students and their parents to test age appropriateness and comprehension of terminology. This informed our measurement development for the 2017-2018 pilot survey, which was iteratively adapted based on multiple data collections at diverse pilot middle school sites. In 2019, we launched a longitudinal survey study based on extensive piloting conducted in a previous study with brand new middle school sites. We used data from wave 2 of this ongoing data set collected from October to December 2019. The data set was collected using the Qualtrics Qualtrics (Qualtrics, Provo, UT) software. Surveys were optimized for both computers and mobile devices.

Overall, 4 middle school sites (3 public and 1 private) of varying sizes and diverse demographics within urban and suburban areas of the Northeastern United States agreed to participate in a study of social media use and health for fifth to ninth graders. This region of the United States is politically and socially tolerant of sexual minorities, as evidenced in the history of legalizing gay marriage in the region and the recent regional policy review conducted by the Movement Advancement Project of the Transgender Law Center [61]. The original survey was in English and was translated into Spanish and Portuguese at the request of the participating schools because of the demographics of the region to improve reading comprehension for their English-language learning student populations. Trained research assistants proctored web-based surveys in person during a designated advisory or break period during an in-school or afterschool session. Parents could opt out their children from participating in the study. Students were told that the study was voluntary, and they could choose not to take the survey, which would not affect their academic standing either way. Their answers would be kept confidential, and they did not have to answer any questions they found uncomfortable. Students provided web-based assent before participating in the survey. Participation rates ranged from 42% in the afterschool programs to 94.8% in the whole school data collection, including 5.2% parent or student opt-out or absence on the day the survey was administered. The total response rate across sites was 84.5%. All students were given embossed pens whether they participated or not, and the names of students who completed the survey were entered into a raffle prize drawing to win a US $25 gift card incentive at each school site for each day of data collection. School sites were provided with a small honorarium to provide access to their students. A total of 1033 students completed the survey between October and December 2019. The participant descriptions have been provided in Table 1.

**Table 1:** Participant demographic data (N=1033).

Characteristic			Participants
**Age (years)**			
	Value, range	10-16	
	Value, mean (SD)	12.7 (1.21)	
**Gender, n (%)**			
	Female	513 (49.6)	
	Male	505 (48.9)	
	Other	15 (1.5)	
**Sexual minority (yes; n=873 with known sexual orientation), n (%)**			212 (24.3)
	Not sure or questioning	109 (51.4)	
	Attracted to both sexes	41 (19.3)	
	Not attracted to either sex	23 (10.8)	
	Attracted to the same sex	19 (8.9)	
	Other	20 (8.9)	
**Grade, n (%)**			
	Fifth	19 (1.8)	
	Sixth	261 (25.3)	
	Seventh	263 (25.5)	
	Eighth	261 (25.3)	
	Ninth	222 (21.5)	
	Missing information	7 (0.7)	
**Race or ethnicity, n (%)**			
	White	484 (46.9)	
	Hispanic	183 (17.7)	
	Black	102 (9.9)	
	Asian American	68 (6.5)	
	Multiracial	48 (4.6)	
	Native American	18 (1.7)	
	Other	98 (9.5)	
	Missing information	37 (3.6)	
Free or reduced-price lunch eligibility, n (%)			232 (22.5)

### Measures

#### Sexual Orientation

Sexual attraction was used to categorize students as either heterosexual or sexual minorities. Students who reported that they were attracted to the opposite sex were categorized as *heterosexual* (0) and those reporting they were attracted to both sexes, the same sex, not sure or questioning, other, or not attracted to either sex were categorized as *sexual minorities* (1).

#### Types and Motivations for Social Media Websites Joined

##### Social Media Platform Use

Students reported which social media websites they had joined. On the basis of our pilot studies of websites currently popular among middle school students, we included the following options: Snapchat, Instagram, YouTube, Facebook, Tumblr, TikTok, Facebook, Twitter, Pinterest, Reddit, House Party, Discord, Steam, ooVoo, Zepeto, WhatsApp, Kik, Twitch, and VSCO. Students who reported joining one of these websites were assigned a score of 1 (yes), and those who did not join these websites were assigned a score of 0 (no).

##### Motivation for Social Media Use

Students were asked about the reasons for joining social media. Responses included finding a place to express themselves, sharing things with friends, seeing what everyone was posting, and using social media to make new friends. Students were allowed to check all that applied. If a student selected a reason, they were coded as 1 (yes), and if they did not select a reason, they were coded as 0 (no).

##### Second Instagram Status

Students who joined Instagram were asked whether they had a second Instagram page or a *fake Finsta*. For students who reported having a *Finsta*, a follow-up question asked why they had more than one page. Reasons included “I want to share a different side of myself to some of my friends,” “I only want some people to know how I really feel,” “I want to be funny with my friends,” and “I don’t want my family to see what I post.” Students were allowed to check all that applied. If a student selected a reason, they were coded as 1 (yes), and if they did not select a reason, they were coded as 0 (no).

### Social Media Settings and Support System

#### Communication About Sexual Identity

Students categorized as sexual minorities were asked a follow-up question about who they talked about their sexual orientation. Students were asked to check all that applied. Responses included no one, LGBTQ club or group, parents, family members, friends, and people they do not know on the internet. Students who reported talking to one of these people were assigned a score of 1 (yes).

#### Privacy Settings

Privacy settings were measured using a single item asking students to report whether the settings for their favorite social media website were set as *public* (1), *private* (2), or they *don’t know* (3). *Don’t know* responses were coded as missing data.

#### Web-Based Curation

For social media behaviors, students were asked to report whether they did any of the following on their favorite social media website: “Posted updates, comments, photos or videos that you later regret sharing,” “Deleted comments that other have made on your page,” “Posted fake or false information,” “Deleted or block people from your network or friend list,” or “Removed almost all prior posts or deactivated an account.” Students were allowed to check all that applied. If a student selected a behavior, they were coded as 1 (yes), and if they did not select any behavior, they were coded as 0 (no).

#### Web-Based Network Size

The number of friends was measured using a single item “Thinking about your favorite social media site, about how many friends do you have on that site?” Responses ranged from *Less than 50* (1) to *Over 1000* (6).

### Participating in Web-Based Social Support

A total of 3 individual items, responding positively when friends share good news, trying to make friends feel better when sharing bad news, or joining a web-based group to feel less alone, were used to measure how often students used social media as a support system. These items ranged from *Never* (1) to *Always* (5). An open-ended response was requested for those indicating that they joined a web-based group to feel less alone.

### Composition of Web-Based Networks

Students reported who they were friends with or who they followed on social media. They were allowed to check all that applied. Responses included parents, siblings or cousins, other family members, classmates or friends from school, other known adults, celebrities, and people they had never met. If a student selected a friend type, they were coded as 1 (yes), and if they did not select any friend type, they were coded as 0 (no).

### Risky Behaviors on Social Media

#### Parent Disapproval of Joining Social Media Websites

Students were asked whether they had joined a social media website their parents would not approve of (yes or no). If they answered *Yes*, they were provided a follow-up question about the reasons why their parents would disapprove of their website. The forced choice options derived from our pilot studies, included “I am too young,” “I am not ready to handle it emotionally,” “I would spend too much time on it,” “My parents would have limited control over it,” and “They don’t want me to have bad influences.” Participants were also able to write additional reasons.

#### Risky Peer Web-Based Behavior

Students reported the content of social media posts and websites they had seen in the past 12 months, including ways to be thin, hate messages, drugs or alcohol, or self-harm. Students checked all that applied. A value of 1 (yes) was assigned to any student who selected that content, whereas nonselection was assigned a value of 0 (no).

#### Web-Based Peer Harassment

Adapted from a web-based victimization scale developed by Ybarra et al [62], this scale asked participants how often their web-based peers made rude or mean comments, spread rumors on the internet, or were hurt by someone excluding them on the internet. These three items were reported on a 4-point scale ranging from 1 (*Never*) to 4 (*Often*; Cronbach *α*=.77).

### Mental Wellness

#### Loneliness

A 3-item loneliness scale, the revised University of California, Los Angeles Loneliness Scale [63], was used as a brief measure of loneliness for young respondents, which has been shown to be internally consistent with both discriminant and concurrent validity compared with the full measure. The 3-item scale asks, “How often do you feel...” (1) that you lack companionship, (2) left out, and (3) isolated from others (Cronbach *α*=.80). These items used a 3-point response scale: *Hardly ever* (1), *Some of the Time* (2), and *Often* (3).

#### Depressive Symptoms

Depressive symptoms were measured using the Center for Epidemiological Studies-Depression (CES-D) 10-item scale [64], which has been shown to have strong internal consistency, test-retest reliability, convergent validity, and divergent validity. Participants were asked to indicate how often they felt this way during the past week. Items were rated on a 4-point response scale format ranging from *rarely or less than 1 day* (1) to *All of the time 5-7 days* (4; Cronbach *α*=.75). Items were summed, and scores of 10 or above were assigned a value of 1; students with scores less than 10 were assigned a value of 0. A score equal to or greater than 10 was considered depression.

#### Self-harm

The frequency of self-harm was measured using a single item about whether the student had ever tried to harm themselves, eg, cutting, hitting, poisoning, deliberate risk-taking, etc. This was a dichotomous variable of either *Never tried* (0) or *Tried once or twice* (1).

### Data Analysis

Regression analyses were conducted to understand the associations between sexual orientation and social media settings, uses, behaviors, and mental well-being. Web-based harassment and loneliness were analyzed using a structural equation modeling (SEM) framework using multiple indicators. In these SEM models, the outcomes were treated as latent variables, which allowed us to account for measurement errors within the modeling process. These models were estimated using the maximum likelihood estimation. Dichotomous indicators, such as joining social media websites and reasons for social media use, were analyzed using a logistic regression framework. Continuous or ordinal outcomes, such as responding positively when friends share good news and the number of friends on social media, were analyzed using a linear regression framework. To reduce the risk of omitted variable bias, all models controlled for the effects of age, gender, and mother’s highest level of education.

Of the 1033 students who completed the survey, 873 (84.51%) were categorized into either the heterosexual (661/873, 75.7%) or sexual minority (212/873, 24.3%) groups. A total of 14.7% (128/873) were missing covariate information related to mothers’ education (116/873, 13.3%) or identified themselves as a nonbinary gender (12/873, 1.4%). For SEM models, missing data were handled using the full information maximum likelihood estimation. Before interpreting the results of SEM models, we required the models to meet at least three of the following four criteria regarding fit indices: comparative fit index ≥0.90, Tucker-Lewis fit ≥0.90, root mean square error of approximate ≤0.08, and standardized root mean square residual ≤0.05. Both the web-based harassment and loneliness models met these requirements. Samples were further reduced because of the amount of missing data on the outcomes. Most model sample sizes ranged from 637 to 745, suggesting that 14.7% (128/873) to 27% (236/873) (sample size range of 637-745 out of 873 students) of data were missing because of the outcome. A few models had more missing data since fewer students answered questions related to privacy settings (n=556), having an Instagram page (n=515), and follow-up questions related to Instagram use (n=200).

Open-ended responses to the survey were analyzed using a structured tabular thematic analysis on brief texts [65], where a coder inductively generated initial codes and themes for data segments and a second coder verified codes and discussed final codes until agreement was reached.

## Results

### Types of Social Media Websites Joined

Sexual minorities were 1.5 times more likely to have joined Tumblr (*β*=.44; odds ratio [OR] 1.56, 95% CI 1.13-2.14; *P=*.007) or Steam (*β*=.45; OR 1.57, 95% CI 1.16-2.12; *P=*.003) and 33% more likely to have joined Discord (*β*=.28; OR 1.32, 95% CI 1.00-1.73; *P=*.05), but 50% less likely to have joined TikTok (*β*=−.50; OR 0.61, 95% CI 0.49-0.76; *P*<.001), Snapchat (*β*=−.60; OR 0.55, 95% CI 0.44-0.70; *P*<.001), or VSCO (*β*=−.53; OR 0.59, 95% CI 0.45-0.77; *P*<.001), 40% less likely to have joined House Party (*β*=−.47; OR 0.63, 95% CI 0.50-0.79; *P*<.001), and 14% less likely to join Instagram (*β*=−.51; OR 0.60, 95% CI 0.48-0.76; *P*<.001) compared with heterosexual students. There were no significant group differences in the likelihood of joining YouTube, Facebook, Twitter, Pinterest, Reddit, ooVoo, Zepeto, WhatsApp, Kik, or Twitch (Multimedia Appendix 1).

### Joining Web-Based Communities to Feel Less Lonely

A total of 25.9% (55/212) of the sexual minority participants indicated that they had joined a web-based group to feel less socially isolated. More than half of these participants (29/55, 53%) provided an example web-based group to which they belonged in the open-ended response. Of these 29 responses, 23 (79%) were usable responses. Out of these responses, a thematic analysis [65] revealed that 4 were explicitly related to the LGBTQ community, including the Reddit subgroup LGBT and a location-based, in-person support group for LGBTQ youth of color, with additional web-based opportunities for engagement. Other students wrote about web-based communities focused on fandom interests, such as gaming servers, art-sharing websites, or book discussion groups, whereas others referred to group chats designed to communicate with close friends (responses are shown in detail in Textbox 1).

**Open-ended responses from sexual minority youth: joining web-based communities to feel less lonely.**

**Lesbian, Gay, Bisexual, Transgender, Queer, or Other (LGBTQ+) Specific**

- GLASS
- Groups for lesbian, gay, bisexual, transgender, or other issues or mental health things
- LGBT Reddit forum
- Some LGBTQ+ Instagram accounts

**Shared Interests or Identities**

- A group chat with people who also have Crohn disease
- A reading and writing community just to share books I’ve read and see what other books people liked
- Robotics
- Gacha community
- Roblox group
- Amino
- An art group on Pinterest, The Drawing Crew
- Anime
- Any occasion (eg, Halloween, matching outfits, and help for homework)
- Discord-Dragon Ball Z: Final Stand
- Episode community and fandoms of shows
- Fortnite group chat called THE FORTNITERS
- Littlest pet shop
- Meme team
- Teenager-focused Reddit forum

**Friend Group Chats**

- Best friends group chat
- Just a group chat with most of my friends

### Motivations for Social Media Use

Sexual minorities were 1.5 times more likely to report that a reason for joining social media was finding a place to express themselves (*β*=.45; OR 1.57, 95% CI 1.20-2.06; *P=*.001) and 25% less likely to report joining to share things they enjoy with friends (*β*=−.28; OR 0.76, 95% CI 0.60-0.95; *P=*.02) than heterosexual students. There were no significant group differences in the use of social media to see what everyone was posting or make friends.

Sexual minorities were 50% less likely to report having a *Finsta* to be funny with friends (*β*=−.66; OR 0.52, 95% CI 0.31-0.85; *P=*.01). Heterosexual and sexual minorities did not significantly differ for any other reasons for having a second Instagram page.

### Social Media Settings and Social Support System

Sexual minorities were asked who they talked to about their sexual orientation; 39.1% (83/212) reported talking to no one, 6.6% (14/212) talked to a Gay-Straight Alliance club or LGBTQ support group, 26.9% (57/212) talked to their mothers, 13.2% (28/212) talked to their father, 8% (17/212) talked to a female sibling or cousin, 7.1% (15/212) talked to a male sibling or cousin, 7.1% (15/212) talked to another female family member, and 2.8% (6/212) reported talking with another male family member. A total of 30.7% (65/212) of sexual minorities reported talking to friends, and 7.1% (15/212) reported talking to people they did not know on the internet.

Sexual minorities were 30% less likely to report that their social media privacy settings were set to *Private* (*β*=−.33; OR 0.72, 95% CI 0.54-0.96; *P=*.03) than heterosexual students. Sexual minorities were also less likely to have deleted or blocked people in their network (*β*=−.28; *P=*.03), but heterosexual and sexual minority youth were equally likely to report social media behaviors such as posting things they later regret, deleting comments, posting fake information, or removing previous posts.

Sexual minorities reported having smaller networks on their favorite social media websites (*β*=−.57; *P*<.001), less often responded positively when friends shared good news (*β*=−.35; *P=*.002), and less often tried to make friends feel better when they shared bad news (*β*=−.30; *P=*.01). However, sexual minorities more often reported joining a group or web-based community to make themselves feel less alone (*β*=.28; *P=*.003), unlike heterosexual youth.

Sexual minorities were less likely to include on their social media their parents (mom: *β*=−.26; OR 0.77, 95% CI 0.61-0.98; *P=*.03 and dad: *β*=−.27; OR 0.76, 95% CI 0.59-0.98; *P=*.04), siblings or cousins (*β*=−.49; OR 0.62, 95% CI 0.49-0.78; *P*<.001), classmates of the same or younger (*β*=−.43; OR 0.65, 95% CI 0.52-0.82; *P*<.001) or older (*β*=−.67; OR 0.51, 95% CI 0.40-0.65; *P*<.001) grades, friends from afterschool or a team (*β*=−.57; OR 0.57, 95% CI 0.45-0.71; *P*<.001), or friends of other friends (*β*=−.58; OR 0.56, 95% CI 0.44-0.71; *P*<.001) than heterosexual youth. Sexual minorities were also less likely to follow any celebrities (sports: *β*=−.73; OR 0.48, 95% CI 0.37-0.63; *P*<.001; actors or actresses: *β*=−.48; OR 0.62, 95% CI 0.49-0.79; *P*<.001; fashion or beauty: *β*=−.40; OR 0.67, 95% CI 0.49-0.91; *P=*.01, health or fitness: *β*=−.85; OR 0.43, 95% CI 0.25-0.72; *P=*.001). Heterosexual and sexual minority youth did not differ in their likelihood of being friends with an adult extended family, teachers, coaches, or people they have never met.

### Risky Behaviors in Web-Based Networks

Sexual minorities were 1.5 times more likely to have joined a social media website their parents would disapprove of (*β*=.41; OR 1.50, 95% CI 1.14-1.97; *P=*.004), and they were more likely to report seeing videos on the internet related to self-harm (*β*=.33; OR 1.39, 95% CI 1.06-1.83; *P=*.02) than heterosexual youth. There were no significant group differences in watching videos related to being thin, hate messages, experiences of drugs or drinking, or frequency of web-based harassment.

When invited to share why they thought their parents would not approve of their presence on specific social media websites, many sexual minority youth referenced the proliferation of mature content, whether it came from other users or their own activity on the website. A total of 54% (20/37) of sexual minority youth who answered this question said that their parents would disapprove because they were too young to be using a certain website, whereas 40% (15/37) thought their parents would primarily be concerned with *bad influences* on the web and 19% (7/37) perceived their parents’ concerns as related to their children’s emotional immaturity. Open-ended answers corroborated these concerns, describing fears about people who “say inappropriate stuff” on web and “people you haven’t met...who want to steal my information.” Time was also a significant concern; 35% (13/37) of sexual minority youth who answered this question thought their parents worried about them spending too much time on the internet, with a participant specifically referencing the quantity of time spent playing Fortnite as a reason for their parent’s disapproval. Limited parental control was another facet of parental disapproval, which was identified by 16% (6/37) of sexual minority youth who answered this question and highlighted in open-ended responses.

### Mental Wellness

Sexual minorities had higher averages of loneliness and social isolation (*β*=.19; *P*<.001) than heterosexual students. They were also twice as likely to have tried to harm themselves in the past (*β*=.81; OR 2.24, 95% CI 1.64-3.06; *P*<.001) and more likely to have symptoms that reached the CES-D-based definition of depression (*β*=.15; OR 1.16, 95% CI 1.08-1.25; *P*<.001).

## Discussion

### Principal Findings

Our study is among the first to examine the social media behaviors and mental health patterns of early adolescent sexual minorities aged as young as 11 years when early sexual identity questioning is more prominent and can influence one’s sense of belonging and mental wellness. The first objective of our study was to understand how sexual minority youth use social media differently than their heterosexual counterparts. Contrary to the general finding that Snapchat, Instagram, and TikTok are the most popular social media websites in early adolescence, we found that sexual minorities gravitate to less popular websites, such as Tumblr and Discord, and were significantly more interested in web-based self-expression than their heterosexual counterparts. Sexual minority youth were also less likely to use *Finstas* or *fake Instagram* accounts. In addition, sexual minority youth were more likely to sign up for social media websites that parents would not approve of and were also less likely to include parents in their web-based networks.

Our second objective was to understand the role of internet peers and socially supportive web-based behaviors. Sexual minority youth tended to have significantly smaller web-based networks, which may partly explain why they were less concerned about privacy settings. In terms of social support, sexual minority youth were less likely to unfriend peers or remove previous posts, presumably because of negativity on the internet. At the same time, they were also less likely to offer social support to their web networks. One of our key findings regarding web-based social support was that sexual minorities were significantly more likely than heterosexual peers to report that they join web-based communities to feel less alone.

Our third objective was to examine any mental health patterns that could help explain the differences in social media behaviors. We found that sexual minority youth were more likely to experience loneliness, depressive symptoms, and even acts of nonsuicidal self-harm than their heterosexual counterparts, which underscores the overall pattern of web-based isolation and the continued struggle to find safe outlets for self-expression.

### Types and Motivations for Different Platforms

Our finding that sexual minority youth were 1.5 times more likely to use Tumblr is corroborated by other research that analyzes Tumblr’s design and community structure, which is especially conducive to identity development and privacy management for LGBTQ youth [21,22]. Although Tumblr’s community makeup changed in 2018 as a result of its adult content ban, which particularly affected the LGBTQ community on the website [66], our data (collected a full year after the content ban went into effect) indicate that Tumblr is still a relevant social media platform that sexual minority youth gravitate toward to express themselves—one that offers a community that other popular websites (eg, Instagram and Snapchat) may not at this early adolescent stage. The accompanying finding that sexual minority youth were 50% less likely to use TikTok is surprising, given that journalistic sources have documented blossoming queer communities on TikTok [67,68]. As a new and continuously evolving platform, it is possible that sexual minority youth might have begun to use TikTok differently during the onset of the COVID-19 pandemic, after these data were collected (in October-December 2019). Although TikTok has proven successful in fostering safe spaces in certain cases, its connotations as a website for funny content might limit its utility for sexual minority youth who are primarily looking for sources of self-expression rather than entertainment in their social media use.

In our qualitative responses, the web-based communities sexual minority youth participated in to reduce loneliness and isolation were tailored around social connections based on identity or interests. The community offered by the Reddit subgroup r/LGBT, for instance, centers around information and experience sharing rather than comedic relief. Other responses reflected the tendency of sexual minority to use digital media to engage with a curated network of close friends, usually in the format of a group chat. Although these responses did not address how they might have formed these friendships initially, it can be partially contextualized by findings from the study by Ybarra et al [38] that internet friends offered higher levels of social support for sexual minority youth. The inclusion of a web-based community that is an extension of a place-based group (GLASS, Boston-based service provider for LGBTQ youth of color and their allies) in open-ended responses is also notable, and further evidence of the Fox and Ralston [16] finding that LGBTQ youth may use the internet to connect with local LGBTQ community spaces.

Sexual minority youth prioritizing self-expression over entertainment was further evidenced by their motivations for social media use. Our sexual minority participants reported being more likely to have joined social media for expressive purposes and less likely to have joined to share things they enjoyed with friends. This use pattern is also supported by the lower likelihood of sexual minority youths using Finstas to be funny. Although some research suggests that humor is a primary function of Finstas for many young adults [69], the sexual minority youth that we surveyed were less likely to share funny content. These results suggest that sexual minority youth may enjoy the more reflective and unidirectional aspects of self-expression on the web, such as having an audience to explore different sides of themselves, rather than being a vessel for an interactive exchange of social support.

### Privacy Settings and Social Support

Our unexpected finding of sexual minority youth being significantly less likely to have private social media settings contradicts previous research that describes web-based spaces as places to control information for a *networked public* [25] or fear of being accidentally outed by automated privacy controls [21]. A possible explanation for this result may be that the smaller networks sexual minority youth already maintain on social media limit their need for explicit forms of privacy control; in other words, their intended audience is only their direct friends or followers, and although they may be aware of and concerned about the use or spread of their personal data, they are unmotivated or powerless to navigate the complex privacy structures of different platforms, as described by Pangrazio and Selwyn [26]. They may also be encoding information using methods other than privacy controls—for instance, engaging in the culture of *subtweeting* detailed by Marwick and boyd [25], where Twitter users allude to interpersonal conflict without revealing details or the identities of individual actors.

The findings that sexual minority youth are less likely to report their nuclear family members as friends on their *networked publics* and that they tend to sign up for social media websites that their parents would disapprove of are possible indicators of fear of revealing their web-based identities to their family members. We hypothesize that this could be because of sexual minority adolescents not being comfortable or ready to *out* themselves to their families in terms of what they post and who it reaches. Being more likely to have joined a social media website without parental approval may also indicate that sexual minority adolescents do not have as much close internet supervision. Although our findings did not show that sexual minority youth are exposed to more mature content than their heterosexual counterparts, except for content relating to self-harm, age and appropriateness of content are typically common concerns for parents. This discrepancy between parents’ and adolescents’ understanding of social media use may be explained by the finding that the time adolescents spend on the web displaces the time they could spend communicating with their families about their behaviors on the web, a pattern that may include sexual identity explorations [70].

Almost 40% of participants in our study disclosed that they had never talked to anyone about their sexual orientation, and two-thirds did not even discuss it with their friends. Given the backdrop of the loneliness and poor mental health outcomes that we uncovered, we also found an overarching pattern of social media use that had a tone of social isolation (eg, not responding when friends share good or bad news and being less motivated to share what they enjoy with friends). These observations may not necessarily be deleterious, as studies have found that having smaller web-based networks or tighter privacy controls to be protective against negativity on the web [71] and having family members find out about identity prematurely when sexual minorities are in the coming out phase may limit youth’s ability to explore their emerging sexual and gender identities. From a different perspective, our findings demonstrate that sexual minority youth have smaller networks, do not share what they enjoy with their internet friends, and are less socially supportive on the internet, all signal forms of passive viewing and disengagement with at least some aspects of social media. According to the minority stress theory, adaptive coping mechanisms may be at play, either through engagement (eg, responding to negativity) or disengagement (eg, avoiding negative spaces) [72]. Active engagement forms of coping often lead to a greater sense of control, whereas disengagement coping can lead to heightened depressive symptoms in offline contexts [73]. Previous research has also established that passive viewing and noninteractive browsing (eg, *lurking*) of social media walls are associated with poor well-being outcomes [74]. An alternative explanation is that sexual minority youth may want to avoid web-based drama by limiting their interactions with a select trusted few [71].

### Risky Behaviors, Mental Wellness, and Harassment

Our study demonstrated significant mental wellness disparities in sexual minorities, such as depressive symptoms, loneliness, and self-harm, in the understudied early adolescent years when they are just exploring their sexual identity statuses. Given the dearth of studies relating sexual minority adolescent social media use and mental health, we conducted exploratory analyses of their web-based behaviors at this stage, including how, why, and with whom they interact on the web, in hopes of understanding any notable preliminary patterns to explore in a future study. For instance, we found that sexual minorities saw significantly more posts about self-harm in their peers’ posts compared with their heterosexual counterparts. We do not know which peers were posting (ie, whether they were a sexual minority peer or not), and we cannot ascertain whether sexual minorities were better able to recognize and remember these types of posts than their heterosexual counterparts. However, the finding that early adolescents significantly view more web-based posts about self-harm and are also significantly more likely to have ever self-harmed is a cause for concern and should be investigated in future studies.

Surprisingly, our study did not corroborate previous research that sexual minority youth are harassed on the web at higher rates than their heterosexual peers [4,38,75]. A reason is that there is possibly more social acceptance of sexual minorities in this generation, given the relatively high proportion of middle school students who identified as sexual minority. Another explanation could be that the sexual minorities in our sample were less willing to disclose this information. Researchers have found that heterosexual individuals are more likely than their sexual minority counterparts to tell their parents and school staff about cyberbullying incidents, partly because sexual minority youth are significantly more afraid of their technology privileges being taken away compared with their heterosexual peers [47]. Our participants also reported low rates of cyber-victimization overall (Multimedia Appendix 1), which may relate to the setting of our study being school-based rather than a convenience sample of LGBTQ-identified youth, which would more likely be self-selected.

### Limitations

The strengths of this cross-sectional data set include racial, ethnic, and socioeconomic diversity and a relatively high number of participants identifying as sexual minority, as it was a highly representative school-based sample of several communities across the New England area. Limitations include the lack of potential generalizability beyond this region of the Northeastern United States. The study did not include gender minority participants as a comparison category because of the low prevalence in our sample (12/873, 1.4%). A recent study using the National Youth Risk Behavior Survey found that bisexual adolescents were more cyberbullied than gay or lesbian adolescents [76]. This could be another alternative explanation for our relatively low cyber-victimization findings, as we had a low proportion of bisexually identified participants, and we were unable to disaggregate our findings by category of sexual minority in our sample. Although previous samples of sexual minority adolescents have been larger, our sample is unique in that it is younger than these other samples (eg, ages 13-18 years and 14-29 years), with our participants averaging 12.7 years. Given that several measures were developed for this study, limitations in establishing the reliability and validity of such measures are acknowledged. For the items measuring privacy of favorite social media websites, participants were only allowed to indicate public, private, or do not know responses, which would be appropriate for some websites with a binary option but may not completely account for websites with partial privacy options.

### Conclusions and Future Directions

Access to online social support systems is critical for sexual minority youth, who often experience discrimination from offline service providers because of their sexual minority status [77]. This potential barrier is particularly true for sexual minority youth living in nonurban areas or those who fear being outed by being seen at LGBTQ organizations. Given the ability to selectively choose who you are *out to* on the web, mental health professionals working with sexual minority youth who express fear of stigmatization or violence for coming out in their offline lives may consider exploring with whom their clients feel comfortable and can turn to online social support. As part of these considerations, further research on new and evolving digital media platforms, such as Discord and TikTok, could aid providers.

Future longitudinal studies could determine any bidirectional influences of mental well-being and social media use in early adolescent sexual minorities. Future studies that include larger samples of early adolescent sexual minorities would allow for analyzing the subgroups of sexual identity separately and distinguishing sexual orientation from gender identity when reporting results. Although this study did not examine data collected from gender nonbinary or nonconforming youth, research is necessary to explore the similarities and differences in their social media use and mental health compared with sexual minorities, especially in early adolescence when peer acceptance is crucial. More research on parental supervision of sexual minority adolescent social media use may unravel why these adolescents tend to avoid *public* social media audiences that include their own parents and siblings while also not using available privacy settings. Much research on peer influence effects through social media has centered on risk behaviors; therefore, more research on the positive spread of resilient behaviors in sexual minority web-based networks is also needed.

## Appendix

Multimedia Appendix 1 Regression results by sexual orientation.

## Abbreviations

LGBTQ: lesbian, gay, bisexual, transgender, or queer
OR: odds ratio
SEM: structural equation modeling
