# Supplementary material for: Young Sexual Minority Adolescent Experiences of Self-expression and Isolation on Social Media: Cross-sectional Survey Study
Source: JMIR Ment Health. 2021 Sep 15;8(9):e26207. doi: 10.2196/26207 (PMC8482247; doi:10.2196/26207)
Supplement: Multimedia Appendix 1 [file mental_v8i9e26207_app1.docx]

Appendix 1. Regression Results by Sexual Orientation

|  |  | Sexual Minorities | Heterosexual |  |  |  |  |
| --- | --- | --- | --- | --- | --- | --- | --- |
| Variables | | Percent/ Mean (SD) | Percent/ Mean (SD) | B | SE | OR | *P* |
| **Social media sites joined** | |  |  |  |  |  |  |
|  | Instagram | 48 | 75 | -0.15 | 0.06 | 0.86 | 0.01 |
|  | Snapchat | 52 | 77 | -0.60 | 0.12 | 0.55 | <0.001 |
|  | YouTube | 85 | 92 | -0.28 | 0.15 | 0.76 | 0.06 |
|  | Facebook | 15 | 19 | 0.02 | 0.14 | 1.02 | 0.92 |
|  | Twitter | 22 | 28 | 0.02 | 0.13 | 1.02 | 0.91 |
|  | Pinterest | 30 | 31 | 0.05 | 0.12 | 1.05 | 0.70697 |
|  | Reddit | 16 | 16 | 0.25 | 0.16 | 1.29 | 0.1107 |
|  | Tumblr | 11 | 7 | 0.44 | 0.16 | 1.56 | 0.007 |
|  | House Party | 30 | 50 | -0.47 | 0.12 | 0.63 | <0.001 |
|  | Discord | 22 | 19 | 0.28 | 0.14 | 1.32 | 0.047 |
|  | Steam | 15 | 13 | 0.45 | 0.15 | 1.57 | 0.003 |
|  | ooVoo | 6 | 9 | -0.05 | 0.18 | 0.95 | 0.7879 |
|  | Zepeto | 7 | 3 | 0.38 | 0.21 | 1.46 | 0.070 |
|  | Tik Tok | 51 | 66 | -0.50 | 0.12 | 0.61 | <0.001 |
|  | WhatsApp | 26 | 27 | -0.09 | 0.12 | 0.92 | 0.4985 |
|  | Kik | 5 | 7 | 0.03 | 0.18 | 1.03 | 0.8768 |
|  | Twitch | 23 | 29 | -0.03 | 0.13 | 0.97 | 0.8215 |
|  | VSCO | 12 | 29 | -0.53 | 0.14 | 0.59 | <0.001 |
| **Motivations for social media use** | |  |  |  |  |  |  |
|  | To find a place to express myself | 21 | 13 | 0.45 | 0.14 | 1.57 | 0.001 |
|  | To share things I enjoy with my friends | 47 | 60 | -0.28 | 0.12 | 0.76 | 0.017 |
|  | To see what everyone was posting | 30 | 42 | -0.21 | 0.12 | 0.81 | 0.09 |
|  | To make new friends | 18 | 14 | 0.24 | 0.14 | 1.27 | 0.08 |
|  | Do you have more than 1 Instagram page? | 41 | 39 | 0.11 | 0.16 | 1.12 | 0.48 |
|  | I want to share a different side of myself to some of my friends. | 34 | 39 | 0.16 | 0.25 | 1.18 | 0.52 |
|  | I only want some people to know how I really feel. | 20 | 17 | 0.15 | 0.32 | 1.16 | 0.65 |
|  | I want to be funny with my friends. | 22 | 53 | -0.66 | 0.26 | 0.52 | 0.01 |
|  | I don't want my family to see what I post. | 7 | 8 | 0.02 | 0.34 | 1.02 | 0.96 |
| **Social media settings and support system** | |  |  |  |  |  |  |
|  | Private privacy settings | 61 | 75 | -0.33 | 0.15 | 0.72 | 0.03 |
|  | Posted updates, comments, photos or videos that you later regret sharing | 19 | 21 | 0.09 | 0.15 | 1.10 | 0.54 |
|  | Deleted comments that others have made on your page | 16 | 18 | -0.06 | 0.15 | 0.94 | 0.68 |
|  | Posted fake or false information | 4 | 2 | 0.32 | 0.28 | 1.38 | 0.24 |
|  | Deleted or block people from your network or friend list | 37 | 51 | -0.28 | 0.13 | 0.76 | 0.03 |
|  | Removed almost all prior posts or deactivated an account | 14 | 18 | -0.06 | 0.18 | 0.94 | 0.72 |
|  | Number of friends on favorite social media site | 2.20 (1.47) | 2.95 (1.54) | -0.57 | 0.15 |  | <0.001 |
|  | Respond positively when friends share good news | 3.42 (1.41) | 3.70 (1.22) | -0.35 | 0.11 |  | 0.002 |
|  | Make friends feel better when they share bad or sad news | 3.42 (1.52) | 3.69 (1.32) | -0.30 | 0.12 |  | 0.01 |
|  | Joined an online community that made you feel less alone | 1.64 (1.17) | 1.43 (0.99) | 0.28 | 0.09 |  | 0.003 |
| **Friends on social media** | |  |  |  |  |  |  |
|  | Your mom(s)/female guardian(s) | 28 | 38 | -0.26 | 0.12 | 0.77 | 0.03 |
|  | Your dad(s)/male guardian(s) | 17 | 26 | -0.27 | 0.13 | 0.76 | 0.04 |
|  | Siblings or cousins | 50 | 70 | -0.49 | 0.12 | 0.62 | <0.001 |
|  | Aunts, uncles, or grandparents | 25 | 31 | -0.17 | 0.12 | 0.85 | 0.18 |
|  | Classmates in your grade or younger | 49 | 72 | -0.43 | 0.12 | 0.65 | <0.001 |
|  | Classmates in upper grade | 30 | 62 | -0.67 | 0.12 | 0.51 | <0.001 |
|  | Teachers or coaches | 3 | 5 | -0.27 | 0.23 | 0.77 | 0.25 |
|  | Friends from after school activity or team | 39 | 67 | -0.57 | 0.12 | 0.57 | <0.001 |
|  | Friends of my friends | 37 | 62 | -0.58 | 0.12 | 0.56 | <0.001 |
|  | Sports celebrities | 15 | 42 | -0.73 | 0.14 | 0.48 | <0.001 |
|  | Favorite actors/actresses | 24 | 41 | -0.48 | 0.13 | 0.62 | <0.001 |
|  | Fashion or beauty bloggers | 12 | 22 | -0.40 | 0.16 | 0.67 | 0.01 |
|  | Health and fitness bloggers | 3 | 12 | -0.85 | 0.27 | 0.43 | 0.001 |
|  | Other people you have never met in person | 23 | 33 | -0.16 | 0.13 | 0.85 | 0.22 |
| **Risky behaviors on SM** | |  |  |  |  |  |  |
|  | Have you ever joined a social media site that your parents would not approve of? | 18 | 11 | 0.41 | 0.14 | 1.50 | 0.004 |
|  | Viewing posts about... ways to be very thin | 14 | 14 | -0.03 | 0.14 |  | 0.84 |
|  | Viewing posts about...  hate messages that attack certain groups or individuals | 21 | 21 | 0.21 | 0.13 |  | 0.11 |
|  | Viewing posts about... sharing experiences of taking drugs or drinking | 16 | 19 | 0.13 | 0.14 |  | 0.35 |
|  | Viewing posts about... ways of physically harming or hurting themselves | 17 | 11 | 0.33 | 0.14 |  | 0.02 |
|  | Online peer harassment | 1.48 (0.68) | 1.51 (0.63) | 0.04 | 0.06 |  | 0.48 |
| **Mental wellness** | |  |  |  |  |  |  |
|  | Loneliness scale | 1.58 (0.57) | 1.41 (0.51) | 0.19 | 0.04 |  | <0.001 |
|  | Depressive symptoms (dichotomous, >score of 10) | 33 | 19 | 0.15 | 0.04 | 1.16 | <0.001 |
|  | Self-harm (tried once or twice) | 14 | 4 | 0.81 | 0.16 | 2.24 | <0.001 |

OR=Odds Ratio, B=Standardized coefficient.
